# Brenner Tumor of the Ovary in a Patient With Postmenopausal Bleeding: A Case Report

**DOI:** 10.7759/cureus.67753

**Published:** 2024-08-25

**Authors:** Kareena Sagar, Pierre F Lespinasse, Ashley Haney, Nicholas B Conway

**Affiliations:** 1 Department of Obstetrics, Gynecology & Reproductive Health, Rutgers University New Jersey Medical School, Newark, USA

**Keywords:** estrogen secretion, endometrial carcinoma, brenner tumour, postmenopausal bleeding, adnexal mass

## Abstract

Brenner tumors are ovarian epithelial tumors that can be benign, borderline, or malignant. This report highlights a case of a patient with postmenopausal bleeding and elevated estradiol associated with a Brenner tumor. A 59-year-old woman, menopausal for seven years, presented with postmenopausal bleeding for the past month. An ultrasound done four years prior to presentation revealed a right adnexal mass likely to be a fibrous lesion. An office endometrial biopsy done at the time of presentation showed a weakly proliferative endometrium. The patient was then prescribed a course of medroxyprogesterone acetate therapy. Because of persistent bleeding, the patient was scheduled for a hysteroscopy and dilation and curettage. An exam under anesthesia confirmed a firm, palpable mass in the right adnexa and a normal uterine cavity. Endometrial curetting indicated proliferative endometrium. After hysteroscopy and biopsy, a pelvic sonogram showed a 5.8 x 4.3 x 4.2 cm solid right adnexal mass. Serum estradiol was measured at 137.0 pg/mL. The patient was then scheduled for a laparoscopic hysterectomy with bilateral salpingo-oophrectomy, with final pathology of the right adnexal mass revealing a Brenner tumor. The patient had an uncomplicated postoperative course. Patients with persistent postmenopausal bleeding require further evaluation; if not caught early, postmenopausal estrogen production by tumors may be associated with concomitant endometrial cancer.

## Introduction

In evaluating a patient with postmenopausal bleeding, the American College of Obstetricians and Gynecologists (ACOG) recommends prompt evaluation either through transvaginal ultrasound or endometrial sampling to exclude endometrial carcinoma and endometrial intraepithelial neoplasia [1]. Vaginal bleeding is the primary presenting symptom in approximately 90% of postmenopausal individuals with endometrial carcinoma and is an even more concerning symptom when the patient presents with risk factors for endometrial cancer, including age, obesity, use of unopposed estrogen, or a family history of gynecologic malignancy [1,2]. While transvaginal ultrasound can assist in detecting a thickened endometrium, a definitive diagnosis is confirmed via endometrial biopsy [1].

Studies have found that estrogen, when unopposed by progesterone, is associated with an increased risk of endometrial hyperplasia [3]. Moreover, the type I subtype of endometrial carcinoma is thought to develop from an estrogen-driven pathway starting at atypical endometrial hyperplasia and is correlated with elevated serum estrogen and a high body mass index [4]. Patients presenting with postmenopausal bleeding secondary to proliferative endometrium should be investigated for some endogenous or exogenous source of estrogen. Beyond the production of estrone by peripheral fat, some functional ovarian tumors are known to secrete hormones [5]. The majority of functional ovarian tumors originate from stromal or sex cord tissue [5]. The ovarian stroma produces androgens; therefore, tumors such as thecomas and stromal luteomas originate from stromal tissue and are associated with androgen secretion [5]. These androgens can then be converted to estrone by the aromatase-containing adipose cells of the peripheral fat [6]. Other ovarian tumors, such as granulosa cell tumors, originating from the sex cords, are known to produce estrogen and inhibin and should be suspected in women with an adnexal mass and postmenopausal bleeding [7,8].

Ovarian epithelial tumors, derived from different types of epithelial lineages in the female reproductive tract, are the most common type of neoplasms that occur in the ovaries [9]. They are classified as benign, borderline, or malignant based on their histologic features; most adnexal masses are benign and found incidentally [8,10]. The likelihood of malignancy increases with certain risk factors, including increased age and postmenopausal status, family history, nulliparity, early menarche, and late menopause [8]. Certain biomarkers, such as cancer antigen 125 (CA 125), can help distinguish benign from malignant adnexal masses, but the sensitivity and specificity in doing so can range from 61% to 90% and 71% to 93%, respectively [8]. CA 125 is elevated in only half of cases of early-stage epithelial ovarian cancer and rarely in cases of germ cell, stromal, or mucinous cancer [8].

Brenner tumors represent a distinct subtype of ovarian epithelial tumors that are composed of urothelial-type epithelial cells and account for around 2% of all ovarian epithelial neoplasms [9]. Among Brenner tumors, 99% are benign, while the other 1% are classified as borderline or malignant [9]. They are usually diagnosed in patients between the ages of 30 to 79, with the average age of discovery being 50 years old [9].

Benign Brenner tumors are typically described as solid, usually unilateral, ovarian tumors with a smooth external surface that tends to measure less than 2 cm. Borderline and malignant tumors are slightly larger than benign tumors and contain more friable tissue [9]. Histologically, Brenner tumors are composed of nests of ovoid or polygonal cells similar to urothelial cells in a fibromatous stroma [9]. These cells often have nuclear grooves that give them the appearance of “coffee bean” nuclei [9]. While Brenner tumors are generally thought to be hormonally inert, there have been several cases linking Brenner tumors with postmenopausal endometrial hyperplasia [11,12]. It is important to note that Brenner tumors have an excellent prognosis and can be cured with complete excision through either unilateral oophorectomy or total hysterectomy and bilateral oophorectomy [9].

This paper describes an interesting case of a patient with postmenopausal bleeding and elevated estradiol associated with a Brenner tumor.

The following text was previously presented as an abstract at the ACOG District III Junior Fellows Day. It was published in the program’s Abstract Booklet on October 27, 2023, in Philadelphia, Pennsylvania.

## Case presentation

The patient, a 59-year-old woman who has been menopause for seven years, presents with a medical history significant for hypertension, hyperlipidemia, coronary artery disease, previous myocardial infarctions, ischemic cardiomyopathy, left ventricular thrombus, and bilateral renal infarcts, on prasugrel 10 mg daily and aspirin 81 mg daily. Her body mass index (BMI) at the time of presentation was 28.5 kg/m^2^. Past surgical history is significant for multiple cardiac stent placements. Of note, the patient had a computed tomography (CT) scan of the abdomen and pelvis with intravenous (IV) contrast done four years prior to presentation that incidentally revealed a large right ovary for a postmenopausal female. Further transvaginal and transabdominal pelvic ultrasound showed normal left adnexa and a solid 3.4 x 2.7 x 2.8 cm mass in the right adnexal region with appearance characteristic of either an ovarian fibroma or pedunculated fibroid. This study also noted a 2 mm endometrial stripe. Because the mass was less than 10 cm in diameter and characterized as benign on ultrasound in an asymptomatic patient, the decision was made for conservative, non-surgical management of this adnexal mass with close follow-up [13].

Four years later, the patient presented to her routine health maintenance appointment with abnormal uterine bleeding and pelvic pain for the past month. The bleeding initially manifested as mild spotting that became more pronounced the day before she presented. The patient was not on any hormone replacement therapy prior to presentation. On examination, scant dark blood was noted in the vaginal vault. The cervix appeared normal, with a palpable mass within the uterus assumed to be a leiomyoma based on the patient’s history of fibroid disease on a previous ultrasound. Adnexal examination revealed no palpable abnormalities. Laboratory studies from the initial visit aimed to assess a coagulation profile, including the prothrombin time (PT), international normalized ratio (INR), and activated partial thromboplastin time (APTT). The patient's PT was measured at 12.7 sec, INR at 0.9, and APTT at 24.6 seconds, all within the reference range. These values are highlighted in Table 1. These coagulation studies do not indicate any underlying inherent or medication-induced coagulopathy as a cause of this patient’s abnormal uterine bleeding.

**Table 1:** Coagulation profile lab results, including reference ranges. Reference ranges are based on values from University Hospital, Newark, NJ.

Component	Patient lab value	Reference range
Prothrombin time	12.7 sec	12.1–14.8 sec
International normalized ratio	0.9	0.9–1.17
Activated partial thromboplastin time	24.6 sec	24.0–34.2 sec

The ACOG recommends either blind endometrial sampling or a transvaginal ultrasound in the diagnostic evaluation of postmenopausal bleeding [1]. As endometrial biopsy is ultimately required to confirm a diagnosis of endometrial carcinoma, the decision was made to proceed directly with endometrial sampling at the patient’s presenting visit. An in-office endometrial biopsy indicated weakly proliferative endometrium with breakdown. 

The patient was initially prescribed oral medroxyprogesterone acetate therapy at a dosage of 10 mg per day for the management of her abnormal uterine bleeding and remained on hormone replacement therapy for five months. Despite this intervention, the bleeding persisted, prompting further investigation. As per ACOG guidelines, if blind endometrial sampling fails to detect endometrial hyperplasia or malignancy, as observed in this patient's case, persistent bleeding warrants further testing with a hysteroscopy with dilation and curettage [1]. Consequently, the patient underwent a hysteroscopy and dilation and curettage (D&C) five months after her presentation.

During the procedure, the uterine cavity was found to be normal with atrophic endometrium. However, a firm, palpable mass was discovered in the right adnexa during the pelvic exam done under anesthesia. Data have shown that bimanual pelvic examination can have limitations for evaluating the adnexa, especially in patients with obesity or a larger uterus [14]. Although the patient’s pelvic examination at the time of presentation did not reveal an adnexal mass, it is likely that a more thorough exam done under anesthesia helped elucidate the adnexal mass. The histopathological examination of the endometrial curetting revealed findings consistent with proliferative endometrium exhibiting extensive breakdown changes and scattered stromal plasma cells, indicative of chronic endometritis and consistent with the blind endometrial sampling done at the initial visit.

**Figure 1:**
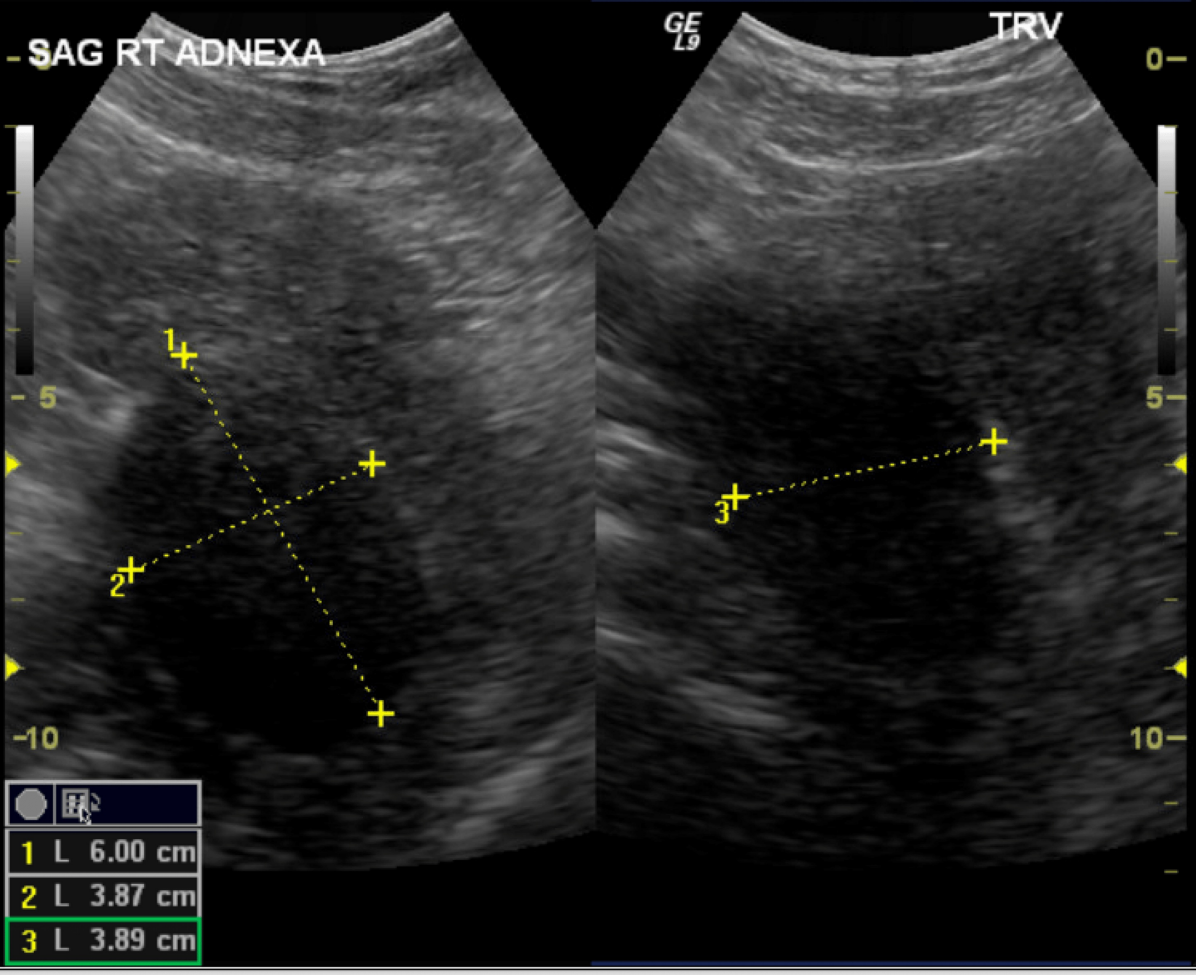
Sagittal view of the right adnexa demonstrating a 6.00 x 3.87 x 3.89 cm mass.

**Figure 2:**
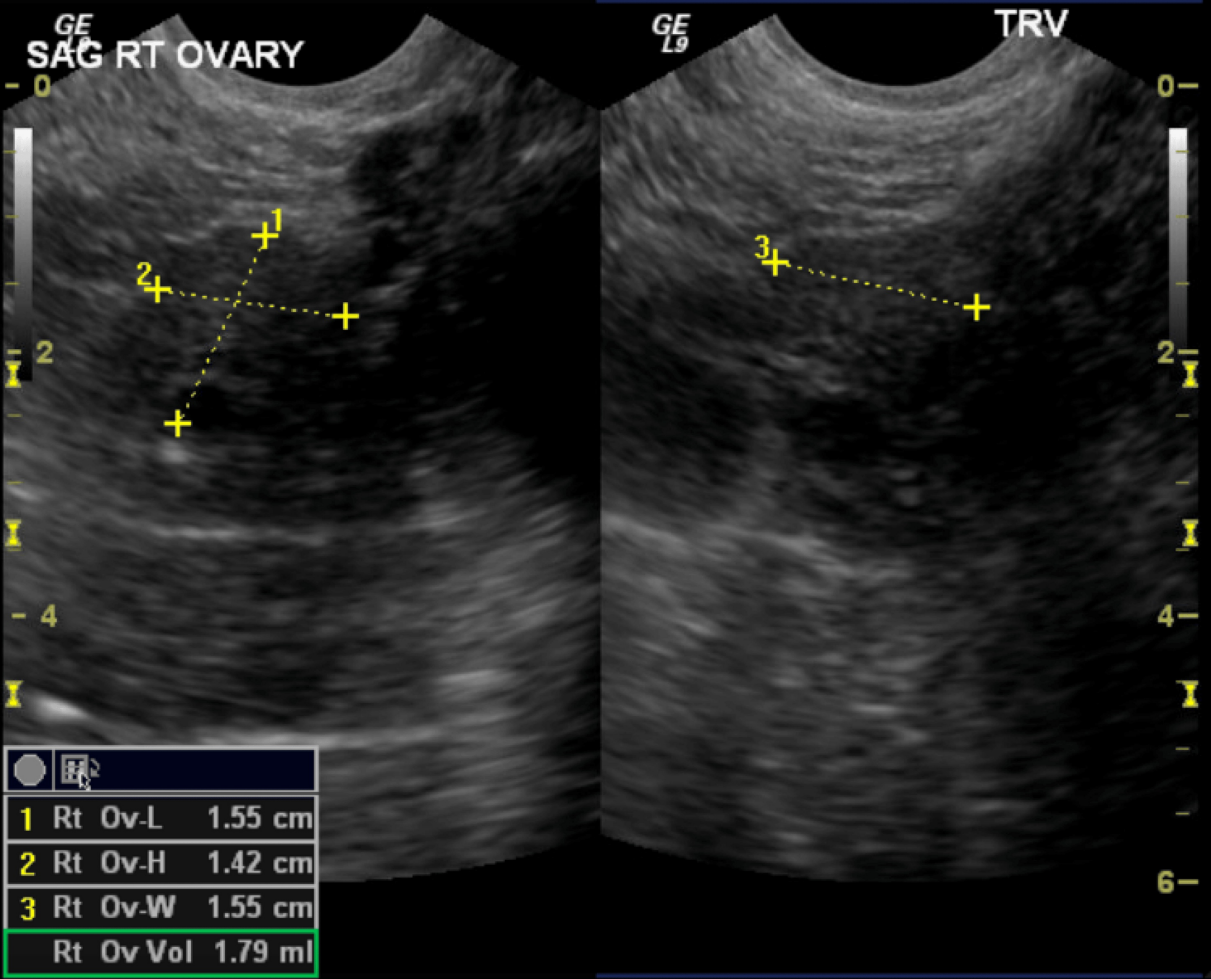
Sagittal view of the right ovary measuring 1.55 x 1.42 x 1.55 cm with an overall volume of 1.79 mL.

**Figure 3:**
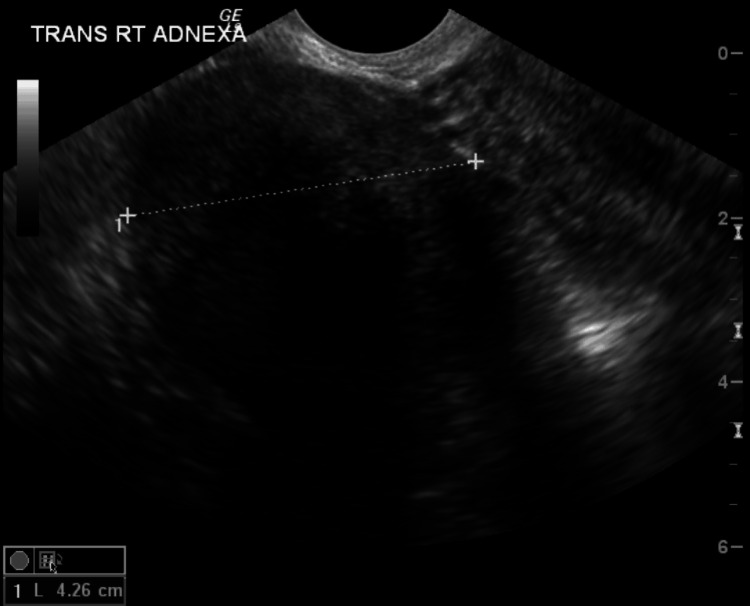
Transverse view of the right adnexal mass measuring 4.26 cm in length.

After the hysteroscopy, the patient underwent transvaginal and transabdominal pelvic ultrasounds to assess the adnexal mass. The ultrasound imaging revealed several noteworthy findings, including a 0.5 x 0.3 x 0.4 cm anterior uterine leiomyoma, an endometrial thickness of 0.4 cm, a left ovary size of 1.0 x 0.8 x 1.0 cm, and a right ovary size of 1.6 x 1.4 x 1.6 cm with a right adnexal mass, as shown in Figures 1-3. The mass was described as a pedunculated, ligamentous fibroid measuring 5.8 x 4.3 x 4.2 cm. This measurement represented an increase in size from a previous study conducted four years prior to presentation, where the mass was measured at 5.1 x 4.1 x 3.8 cm.

Due to this patient’s presentation of postmenopausal bleeding and proliferative endometrium, now with a confirmed adnexal mass, clinical suspicion was high for an ovarian tumor that was likely to be secreting estrogen, most commonly a granulosa cell tumor [8]. Granulosa cell tumors are known to produce both estrogen and inhibin, so serum lab work was taken at this point to assess the patient’s estradiol, follicle-stimulating hormone (FSH), and inhibin B levels [8]. Because CA 125 levels are more closely associated with epithelial ovarian cancer and are not often elevated in sex cord-stromal tumors, it was not indicated to measure CA 125 levels in this patient at this time [8]. Notably, the patient's estradiol level was measured at 137.0 pg/mL, FSH level at 16.6 mIU/mL, and inhibin B level at 15.7 pg/mL. These levels suggest that there was an extrafollicular estrogen source significant enough to suppress circulating levels of FSH below what is normally seen in postmenopausal women [15]. These values were compared against established reference ranges and are detailed in Table 2.

**Table 2:** Lab results, including reference ranges. Reference ranges are based on values from University Hospital, Newark, NJ.

Serum	Patient Lab Value	Reference Range in Postmenopausal Female
Estradiol	137.0 pg/mL	˂6.0 – 54.7 pg/mL
Follicle-stimulating hormone	16.6 mIU/mL	26.7 – 133.4 mIU/mL
Inhibin B	15.7 pg/mL	0.0 – 16.9 pg/mL

Following the diagnostic assessments, the patient was scheduled for a laparoscopic hysterectomy with bilateral salpingo-oophrectomy. Frozen sections taken during the procedure were suggestive of a benign disease process, so the decision was made to forgo lymph node resection at that time. The surgical procedure resulted in a final specimen of the uterus, fallopian tubes, and ovaries weighing 254 g and measuring 9.5 x 5.2 x 3.2 cm, with the endometrium measuring 0.3 cm in thickness. The gross specimen of the right ovary measured 7.2 x 4.9 x 3.1 cm, compared to the left ovary measuring 1.9 x 1.5 x 0.9 cm. Pathological examination of the right ovarian mass revealed a Brenner tumor with negative inhibin immunostaining.

Following the surgery, the patient met all her postoperative milestones and was ultimately discharged home in stable condition later that day. At a follow-up appointment in the outpatient clinic on postoperative day eight, the operative site was found to be intact and healing well and the patient reported near resolution of her vaginal bleeding.

## Discussion

Brenner tumor of the ovary is a solid ovarian tumor of connective tissue origin that is generally asymptomatic [11]. Often discovered incidentally, they may only manifest nonspecific symptoms such as an enlarging abdominal mass if they attain a significant size [9]. This is in contrast with this patient’s atypical presentation of postmenopausal bleeding. On gross evaluation, Brenner tumor resembles a leiomyoma, which can lead to misidentification on imaging, particularly in patients with a history of uterine leiomyomas, as observed in this case [11]. Brenner tumor can only be differentiated from a fibroma histologically by the presence of stratified epithelium, prominent nuclear grooves, and frequent association with a mucinous component [9]. The histology of Brenner tumors may be similar to that of granulosa cell tumors, namely, the papillary architecture and typical “coffee bean” nuclei with nuclear grooves [9]. However, a tissue sample positive for inhibin immunohistochemical staining can distinguish the tumor as a sex cord in origin over a Brenner tumor [9,16]. The tissue sample of this patient’s adnexal mass demonstrated negative inhibin immunostaining, making the diagnosis of a Brenner tumor more likely than that of a granulosa cell tumor.

Approximately 4-14% of Brenner tumors have been associated with concurrent endometrial hyperplasia, often accompanied by stromal luteinization and estrogen production, which could account for the abnormal uterine bleeding observed in this patient [12]. One study reported that 11.9% of women with proliferative endometrium on initial endometrial sampling later developed endometrial hyperplasia or cancer, representing a fourfold greater incidence than women with an atrophic endometrium [17]. Given the patient's initial presentation with proliferative endometrium, indicative of estrogen stimulation, there exists a potential risk of progression to endometrial carcinoma, even if the source of estradiol is identified and removed. Failure to excise the tumor could have left her susceptible to concurrent development of endometrial carcinoma, so a timely intervention was crucial to mitigate such risks.

The management of adnexal masses first requires deciding whether the patient should be continually observed or if surgical intervention is necessary [11]. This decision is guided by information taken from the patient’s history, examination, diagnostic studies, and the presence and severity of symptoms [11]. In cases where observation is deemed appropriate, the healthcare team must determine the frequency of follow-up assessments and criteria indicating the need for surgical intervention [11]. Conversely, if surgical evaluation is deemed the most suitable course of action, considerations include whether a specialist's expertise is warranted and whether an open or minimally invasive surgical approach is optimal [11].

Given the patient’s persistent bleeding refractory to medical management, surgical intervention was deemed necessary, especially given the risk of endometrial proliferation progressing to endometrial carcinoma. A hysterectomy was planned to facilitate the removal of the ovarian mass through the vagina since the solid nature of ovarian tumors can make laparoscopic removal through the abdomen difficult. In addition, bilateral salpingo-oophorectomy was chosen over a unilateral approach because the patient was postmenopausal and to remove any possible small, estrogen-secreting tumors on the contralateral ovary.

## Conclusions

A Brenner tumor of the ovary, although uncommon and typically benign, can often be misidentified as an ovarian fibroma and can be overlooked in the workup of a patient. Individuals presenting with postmenopausal bleeding must undergo a thorough assessment for endometrial carcinoma and identification of any potential sources of estradiol secretion. Particularly in cases of ovarian tumors, especially those known to produce estrogen, concurrent evaluation for endometrial carcinoma is essential to ensure comprehensive patient care and management.
